# The perception and interpretation of malaria among Chinese construction workers in sub-Saharan Africa: a qualitative study

**DOI:** 10.1186/s12936-023-04739-4

**Published:** 2023-10-10

**Authors:** Li Zou, Haohao Ma, Mohammad Shahir Sharifi, Wenyu Deng, Xiaoyu Kan, Junfei Luo, Yin Bai, Yunling Ouyang, Wenjuan Zhou

**Affiliations:** 1 School of Humanties, Central South University, Changsha, Hunan Province China; 2 School of Media and Communication, Shanghai Jiao Tong University, Shanghai, China; 3 Insurance Professional College, Changsha, Hunan Province China

**Keywords:** Malaria, Chinese workers, Sub-Saharan Africa, Qualitative study

## Abstract

**Background:**

Cooperation between China and Africa is deepening, and business, trade, and people-to-people exchanges are growing closer together, especially in the infrastructure construction field. At the same time, malaria has become a serious health concern for Chinese construction workers in Africa, who are at increased risk of infection and complications due to lack of immunity and exposure to high-transmission environments. One of the biggest challenges in fighting malaria is their lack of knowledge and misinterpretations about the disease, which can impact their need for interventions, adherence to treatments, and health services. This study aims to determine the perception and interpretation of malaria among Chinese construction workers in sub-Saharan Africa.

**Methods:**

Semi-structured interviews were conducted with 20 Chinese construction workers in sub-Saharan Africa. Some early respondents initially made contact through two Chinese construction companies in Africa, while the rest of the participants were engaged via a snowball method by the early participants. NVivo10, a qualitative research data management software and a thematic approach, was used to analyze the data and create themes. In order to achieve the general study goals, an inductive content analysis was applied.

**Results:**

The study classified participants' perceptions and interpretations of malaria into four categories: flu-like malaria, the rumors of malaria, the hard-to-explain confusion about malaria, and the special interpretation of malaria.

**Conclusion:**

Malaria poses major health issues to Chinese construction workers in sub-Saharan Africa who lack immunity and live in an environment of high transmission. Their dearth of awareness and misunderstanding of malaria impacts their prevention and treatment behaviors and health outcomes. This study adopts qualitative methods to examine their perceptions and interpretations of malaria, which can serve as a source for future health management strategies.

## Background

Despite malaria control improvements, the disease is still a significant public health burden worldwide [1]. Three billion people are at risk of malaria contracting globally every year [2]. According to the World Health Organization's malaria report, there were 241 million malaria cases and 627,000 malaria deaths globally in 2020. This estimated number shows about 14 million extra malaria cases in 2020 compared to 2019, with 69,000 more deaths. Sub-Saharan Africa, a region that has long been the most vulnerable against malaria, shoulders 93% of all malaria deaths globally in 2020 [3].

The number of Chinese workers in Africa has increased for over a decade as a commercial partner to the African region. According to the Chinese Department of Commercial Affairs report (2019–2020), China dispatched 487,500 labour service personnel of various types in 2019, while there were 992,100 Chinese workers abroad at the end of that year [4]. As one of China's commercial partners, Africa received 73,200 Chinese workers of all kinds in 2019, 15% of the total Chinese labour personnel abroad. This number then reached 182,700 by the end of 2019, and most of them were in the infrastructure construction field [4, 5].

As the number of Chinese workers in Africa increases, their malaria risk becomes more important. The Chinese Center for Disease Control and Prevention provides warnings and recommendations for Chinese workers in Africa, as do African local disease control agencies. Moreover, malaria prevention and control for Chinese workers in Africa mainly depends on the companies and employees [6]. As Chinese construction companies in sub-Saharan Africa, the management team is responsible for the implementation, monitoring, and evaluation of malaria public prevention measures, which ultimately include the construction of dormitories with anti-mosquito facilities, the distribution of free mosquito nets, the cleanliness and hygiene of the environment [7]. Project management also reminds employees that they are personally responsible for adopting individual measures, comprising standardizing the application of mosquito nets, lighting mosquito coils, and wearing long-sleeved shirts and trousers/skirts [8], while the management does not enforce adherence at the individual level [9, 10]. However, according to Hui-ming Wu et al., who conducted a survey in Guangzhou airport among Chinese returnees from Africa, the overall malaria incidence rate of this group was 8.98% (134/1492) at high risk, and most of the returnees had experienced malaria infection more than once [11]. According to Lu Lidan et al., who conducted a questionnaire survey in China Guizhou Province on malaria knowledge, attitudes, and behaviors of workers and managers of two Chinese companies focusing on international construction projects, Chinese workers who work in sub-Saharan Africa had malaria-preventative behaviours at a low level of about 40% [11]. In a survey among Chinese construction workers working in Nigeria, Yu Fengting et al. found that only 47.1% of the respondents knew how to treat malaria, 36.4% knew how to prevent malaria, 88.8% of workers visiting Africa were afraid of malaria, and their general awareness is poor [10]. More research is needed, given the severe risk of malaria among Chinese construction workers in Africa.

According to Agius, qualitative research aims to develop concepts that help researchers understand social phenomena in, wherever possible, natural rather than experimental settings to understand individuals' experiences, perceptions, or behaviours and the meanings attached to them [12]. As a result, the practical application of the qualitative method to other disciplines, including clinical, health service, and education research, has rapidly been used. Therefore, a qualitative approach was conducted to determine how the Chinese workers understand malaria.

This study examines Chinese workers' perception and interpretation of malaria in sub-Saharan Africa to propose effective public health interventions to reduce malaria infection rates among Chinese construction workers.

## Methods

### Study population and study sites

This qualitative study was conducted in sub-Saharan Africa, and the data was collected between Feb. 2021 to May 2021. In this study, a snowball method was used to manage the data of Chinese construction workers in sub-Saharan Africa, as this method can help build rapport and trust with the respondents, as their peers recommended. The initial participants were contacted through the two Chinese construction companies in Africa. It is mentionable that, according to the 2020 ENR "World's Largest 250 International Contractors" List, six Chinese construction companies, all state-owned, are leading the African market with a 61.9% market share [13]. They mostly use the same management standard and implement the same malaria preventive measures. Although the mentioned companies win construction contracts individually, they then cooperate in implementing the task. In this study, the two Chinese companies were randomly selected as a bridge to the research subjects. The companies provided ways for the study to access a couple of initial respondents. They were then asked to provide contact information of other respondents who met the following criteria: they must be Chinese, able to communicate in Chinese, have more than one year of work experience in sub-Saharan Africa, have experience in interacting with malaria or malaria patients, and have WeChat (a Chinese cell/web app for messaging or communication) on their smartphones. The participants in this study included warehouse keepers, purchasing agents, human resources, security officers, drivers, logistics, administrators, commercial officers, office clerks, cooks, and labour workers to identify comprehensive malaria perception among the study population. Due to the COVID-19 epidemic, the interview was conducted via a WeChat video conference. Eligible participants were workers who met inclusion criteria and were willing to provide informed consent for participation. Researchers verified eligibility and chose participants to represent the population. Using convenience and purposeful sampling, 20 eligible workers approached and agreed to be interviewed. The research team achieved thematic saturation after 20 interviews and corroborated through discussion and review of critical data.

### Data collection and processing

The study used a semi-structured interview to explore respondents' malaria perception. Interviews were conducted over 14 weeks, from February 2021 to May 2021. The researchers draw up the interview outline based on the literature. Also, to obtain more information related to the topic, we asked some probing questions. The question guide and probing questions have been added in an appendix at the end of the paper. The questions were planned but flexible, so the researchers altered the sequence of questions when required. Respondents were not separated by gender or job due to no noticeable difference in perception. All interviewers and interviewees were Chinese, and the communication language used between them was their native language to remove any language barrier. In this study, 20 online semi-structured interviews lasted 23 h and 4 min were conducted, and 116,216 words of interview materials were obtained. Trained research assistants undertook all the interviews. Due to the pandemic, the interviews were conducted via WeChat, the most popular social media App in China, covering more than 90% of Chinese smartphone users. The researchers bracketed all respondents' feelings while doing the interview. With the respondents' consent, the entire interview was recorded, and each interview lasted no less than 1 h to ensure sufficient information was collected. After completing the interview, researchers timely recorded the scene and related ideas to enhance the credibility of the research. The sample size is based on data saturation when no new participant information appears.

### Data analysis

Data analysis was carried out simultaneously until the data saturation. After the interview, the researchers transcribed the records into text data word by word in 24 h. They provided feedback to the participants via WeChat to verify the authenticity of the content after checking. Finally, NVivo10, a qualitative research data management software, was used for data analysis. Data were organized using a thematic approach in this study. Researchers familiarized themselves with data (through a line-by-line reading of transcripts, memos, and the diary, and through repeatedly relistening to audio files). Finally, codes were organized into themes. The founded themes were discussed regularly among the research team throughout the analytic process. Researchers coded 90% of transcripts independently before comparing analyses, while the discussion resolved differences. To ensure rigor in the data analysis, the researchers employed various strategies. First, researchers used the snowball method to recruit the employees, eventually avoiding researchers' bias of choosing specific respondents and adding reliability to the study. In addition, researchers recruited employees from different positions to make the analysis more comprehensive. Still, the study found no significant differences in their views, so researchers avoided analysing them separately. Second, to add more accuracy to the themes and coding, two researchers conducted coding and constructed themes separately, and the differences were then unified after negotiation. Finally, the study showed membership checks by sharing the findings with participants to ensure the accuracy of the interpretations (Table 1).

**Table 1:** Participants and interview characteristics

Respondent code	Gender	Age	Job	Country	Interview date
A	Male	47	Warehouse keeper	Sierra Leone, Tanzania	Evening, February 12, 2021
B	Male	37	Labour worker	Sierra Leone, Tanzania	Evening, February 14, 2021
C	Male	26	Labour worker	Tanzania	Evening, February 15, 2021
D	Male	42	Purchasing agent	Kenya	Afternoon, February 17, 2021
E	Male	33	Labour worker	Tanzania	Morning, February 19, 2021
F	Female	36	Human resources	Kenya, Tanzania	Afternoon, February 19, 2021
G	Male	39	Cook	Tanzania	Afternoon, March 4, 2021
H	Male	43	Security Officer	Côte d'Ivoire	Evening, March 5, 2021
I	Male	30	Office clerk	Côte d'Ivoire	Afternoon, March 6, 2021
J	Male	51	Driver	Congo	Morning, April 25, 2021
K	Male	54	Logistics	Uganda, Benin, Kenya	Afternoon, April 25, 2021
L	Male	49	Administrator	Uganda, Kenya	Afternoon, April 28, 2021
M	Male	45	Commercial officer	Congo, Uganda	Evening, April 28, 2021
N	Male	36	Labour worker	Cameroon, Gabon	Morning, May 11, 2021
O	Male	31	Labour worker	Kenya	Afternoon, May 11, 2021
P	Female	38	Administrator	Benin	Afternoon, May 13, 2021
Q	Male	29	Labor worker	Côte d'Ivoire, Benin	Evening, May 13, 2021
R	Male	33	Labor worker	Cameroon	Afternoon, May 15 2021
S	Male	49	Administrator	Uganda, Tanzania	Morning, May 16, 2021
T	Male	31	Labour worker	Gabon, Sierra Leone	Afternoon, May 16, 2021

### Ethical considerations

Researchers considered the ethics in recruitment. The Research Ethics Committee of Xiangya School of Medicine, Central South University, approved this study (#E202082). The confirmation of willingness to attend the interview is taken from the participants. The interviewer and all the respondents were Chinese natives, leading to smooth communication.

## Results

### Demographic characteristics of participants

Finally, this study interviewed 18 men and 2 women aged 26–54 who hold various jobs in Chinese construction companies in sub-Saharan Africa.

### Themes

Four themes were found through qualitative data analysis: flu-like malaria, the rumor of malaria, the hard-to-explain confusion about malaria, and the special interpretation of malaria.

#### Flu-like malaria

According to most participants, malaria has the characteristics of "high infectivity, high response efficiency, and low mortality rate," consistent with the epidemic characteristics of influenza. Chinese workers see malaria infection as an inevitable disease in Africa. They showed less fear after the first malaria contract and presumed deaths as exceptional cases. Therefore, they usually consider malaria a "flu-like" disease and underestimate its death risks.

1. "Malaria is almost everywhere here"
  The popularity of malaria infection in Africa led the respondents to perceive that malaria covered a vast, if not all, area of Africa. They have either been infected or have seen numerous infections in their area that are the most common types of malaria. The perception of malaria as an "everyday occurrence" lets them take malaria as nothing out of the ordinary.
  I lived in Congo for a year, and I did some statistics. Basically, one or two in 20 of us get malaria every month. We have a colleague; we all call him "Malaria King." He really gets malaria once every two or three months. (Respondent J).
  Almost all were infected; out of thousands of people who went there, only a few were not infected. If you're feeling unwell, don't ask yourself (if it is malaria), you basically have. Malaria is basically everywhere. (Respondent K).
2. "Malaria is much easier to control after the first infection"
  Repeated infection strengthens immune memory, and symptoms after repeated infection with malaria are alleviated to a certain extent. Moreover, the experience of repeated infections has allowed our respondents to get familiar with malaria symptoms and treatment methods. Misdiagnosis and delayed treatment have become less common among them.
  The first time is the hardest because we have not had such an experience before and feel physically uncomfortable. (Respondent L).
  In fact, our second time was much better, not as much of a problem as the first time. When it comes, let it come. It was really uncomfortable the first time. I didn't have the strength to walk. Once I got through it, it became a lot easier for me because I knew what it looked like. (Respondent A).
  The second time, I said I needed to take this medicine, and it would be fine after one treatment. For the third time last year, it came quickly and recovered quickly. The doctors at the Chinese clinic were all surprised. He said it would take several days to get a percussion shot; how you recovered by just having one shot. I said give me the shot quickly. I have to go back. I'm really fine. (Respondent S).
3. "Deaths from malaria are rare"
  Malaria can threaten the lives of Chinese construction workers. The respondents tend to summarize death cases as "exceptional cases" or attribute them to causes such as COVID-19, heart disease, or other personal reasons.
  This is the only case I noticed during my three years in Africa that died due to malaria. Four people died during my stay there. One may die from COVID-19, and the other two may die from heart problems. (Respondent K).
  One of our colleagues died there of malaria. In fact, his body was relatively weak, and his weak body's resilience caused his death. (Respondent M).

### The rumours about malaria

Rumours are information with poor authentication data. The unverified piece of information that workers have generated after interacting with malaria for many years is the rumours about malaria. The easy-to-spread but hard-to-convince rumours related to malaria have become the perception of most respondents.

1. "Everyone carries malaria parasites"
  The malaria parasite is transmitted to the human host by female mosquitoes of the genus *Anopheles*. It has an incubation period of 7 to 18 days. In some types, it can lie dormant in the human body for a longer time, months, or a year. However, respondents believe anyone bitten by mosquitoes would carry the malaria parasite. Therefore, respondents consider that the malaria parasite carriers stay in the incubation period for even longer and would only have to wait for the attack.
  Many Chinese doctors here told us that as long as people have traveled to Africa, they will all have malaria parasites in their bodies, but you should not be afraid of this disease. (Respondent I).
  People may not feel the malaria parasite in their bodies, but it is a matter of the fact that everyone here carries malaria parasites. (Respondent A)
  When the mosquito bites you, the malaria parasite enters your body. If you do the test, it will show malaria parasites in everyone's body, but it is another matter of whether it attacks or not. (Respondent B).
2. "Less body resilience leads to malaria attack"
  According to scientific knowledge, a malaria attack usually begins after 7 to 18 days of incubation and has shivering, chills, high fever, sweating, fatigue, and other symptoms. The above symptoms start from a feeling of dropping in strength, and when typical symptoms appear, they are recognized as a malaria attack. However, the respondents consider the dropping in "body resilience" as the cause of malaria attacks. They speculate that less resilient people are prone to malaria attacks.
  I think the drop in body resilience leads to the malaria attack issue. When people are tired, their resilience decreases. (Respondent C)
  In general, the elders are vulnerable groups. They have malaria attacks more than other groups. In fact, they get malaria attacks now and then. (Respondent P)
  I observed the infected people around me. They usually stayed up all night and couldn't sleep well. Their mental state was not good. They might have brain fatigue or something for a few days… So, this group of people easily get malaria attacks. ((Respondent G).
3. "Malaria is nothing to be afraid of"
  Malaria is considered one of the most dangerous infectious diseases globally. However, the concept of "not being afraid of malaria" has taken hold among the respondents. This concept can be due to two reasons. First, since workers chose to work in Africa, they have had no choice but to ignore the threats. Second, employers could indirectly encourage workers to work fearlessly, preferring not to highlight the death threats posed by malaria.
  I don't think malaria is terrible, but that doesn't mean you don't have to take it seriously. You just have to treat it in time, and everything will be fine. (Respondent S)
  People like me who know about this disease will treat it as soon as we find out we have malaria performance. It is not a big problem, so we seldom talk about it. (Respondent D)
  If you feel unwell, you should go to the hospital for an examination. No fear. There is no problem if you discover malaria performance early and do the timely treatment. (Respondent H).

### The hard-to-explain confusion about malaria

Alongside the rumored concepts, the study also found widespread confusion. Some confusions have scientific answers but are hard to convince, while some have not been scientifically proven since the history of malaria research.

1. "Why some Chinese workers have never gotten malaria?"
  The existence of exceptional people who receive fewer bits or are never bitten creates confusion for the rest of the malaria interactors. The confusion of why some workers are being bitten more, and some less while in the same living conditions is a tricky question with no scientific answer yet.
  I have a good bro and friend; he has been there for eight or nine years but hasn't been infected once. Let me tell you, he basically ate with us and drank with us. He was also skinny, like the ribs, but had not been infected. I still have no idea why he has not been infected. (Respondent J).
  A friend of mine has a sour stomach and is prone to a bad stomach. He should have poor body resilience, but he has never been infected with malaria. (Respondent I)
2. "Why do I get malaria while I am well protected?"
  The respondents who are well-acquainted with malaria and live in better conditions raise the confusion that they cannot avoid malaria infection while applying the anti-malaria measures.
  You can see that the most authoritative medical expert will also get sick. I pay so much attention to mosquito prevention, and my concept and knowledge are pretty accurate, but I have been infected with malaria four times, and I don't know why. (Respondent L).
  When I go to the port for work at night, I always wear long-sleeved clothes and never wear short-sleeved ones. Look, in my dormitory, I am always using mosquito repellent. With such protection measures, I still have been bitten and infected. (Respondent N).
3. "Whom mosquitoes bite the most?"
  Mosquitoes show a preference for biting human bodies. For instance, body A, which attracts more mosquitoes, will get more bites than body B, which attracts fewer. On the contrary, body A gets more occasional bites when it meets body C, attracting mosquitoes even more. There is no exact scientific answer to the confusion about which body attracts most mosquito bites yet.
  Sometimes, I get bitten a lot; sometimes, it doesn't bite me very much, and it bites others. I don't know whom mosquitoes like to bite. (Respondent F)

### The special interpretation of malaria

Regarding human nature, one tends to generate interpretations for critical cases after an experience. Long-term interaction with malaria in Africa allowed Chinese construction workers to get unique renditions of malaria. In this study, the researchers found two levels of interpretation. One symbolized malaria as the experience of working in Africa, and another linked malaria to predestination.

1. "Who has not been infected with malaria does not count as having been to Africa"
  Respondents generally regarded the experience of malaria infection as a symbol of life in Africa and even considered this experience a sign of "honor" and "medal." The number of infections reflected their work qualifications.
  After a long time, we felt that if you don't get malaria, you don't count as having been to Africa. In fact, if you travel to Africa, you must be infected with malaria to experience this feeling. (Respondent J).
  Someone told me that if you don't have malaria, you're not considered to have worked in Africa. (Respondent C)
2. "Malaria infection is predestined"
  Human nature suggests combining intractable problems with ultimate superfluous power. For various reasons, Chinese construction workers who fail to avoid malaria infection tend to associate malaria with predestination. In addition, according to respondents, having a predestined perception of malaria at a certain level can reduce their mental anxiety, allow them to focus on their work, and keep a good spirit.
  Getting malaria is like getting robbed, depending on how lucky you are. (Respondent G)
  Don't brag about not being infected. Once you say that you're not infected, malaria comes unexpectedly. So, this is a superstition. (Respondent K)
  I think the more anxious you are, the easier it is to get malaria, so I never take it seriously, and I won't think about its threats. I have been here for a year and a half and have never been infected. (Respondent R).

## Discussion

As the number of Chinese construction workers going to sub-Saharan Africa increases, their risk of contracting malaria rises, and their understanding of malaria is thought to influence their risk. This study uses the qualitative approach to examine the perception and interpretation of malaria among Chinese construction workers travelling to sub-Saharan Africa to provide a basis for reducing malaria risk rates in this population.

Due to the widespread prevalence of malaria in sub-Saharan Africa, Chinese construction workers see malaria as a "flu-like" disease with epidemic characteristics such as solid contagion and low mortality. Flu-like illnesses with non-specific symptoms pose big diagnostic problems to clinicians [14], and malaria can cause recurrent or relapsing flu-like symptoms [15]. This finding is similar to some previous studies on local African residents. It is possible that they were African locals who passed this belief on to Chinese workers. In a review, Christopher Pell et al*.* found that residents in many parts of Africa defined malaria as an ordinary and low-risk febrile disease [16]. Like malaria, influenza is a common infectious disease in Africa. However, flu and malaria differ in transmission, diagnosis, and treatment. If malaria is equated with influenza, it will reduce the vigilance of high-risk groups against malaria. It may also delay the diagnosis and treatment of patients [17], resulting in the spread of malaria. Coincidentally, in a study of beliefs about malaria among people of African descent in central London, Morgan and Figueroa-Munoz came across the same finding as the current study did. Their study population distinguished two types of malaria: "a severe cause of death" and a "flu-like" illness. People used to refer to the first as "cerebral" malaria (caused by P. falciparum) and described the "flu-like" malaria as something 'normal' and ordinary [18].

The death rate among malaria infections is relatively low compared to people with the parasite. Therefore, locals used to see malaria as a less threatening disease. Another reason that can cause the strengthening of this opinion amongst Chinese construction workers is the taboo of avoiding discussions about "death." According to Chinese culture and worldview, death is the absolute end for a person, and they prefer not to talk about it. For instance, if ten people die from malaria but one person is rescued, they prefer to talk about the one who recovered and avoid talking about the deaths. Nevertheless, this perception results in underestimating the disease and is not conducive to taking proper preventive measures.

A rumour is a statement or report current without known authority for its truth [19]. It is a proposition or belief passed along from person to person, usually by word-of-mouth, without secure standards of evidence [20, 21]. In public health, it is easy for the public to learn about their health and investigate their health status by obtaining massive amounts of health information. However, if much of this widely circulated information is inaccurate or even outright wrong, people can easily be misled and make wrong health behaviours [22, 23]. In this study, the researchers found some malaria-related rumours. The rumours led Chinese workers to misunderstand malaria. Such as: "everyone carries malaria parasite" and "malaria has nothing to be afraid of." Some rumour was with Chinese characteristics: the decline of the body's immunity will lead to the onset of malaria. The researchers speculate that this may be related to the deep-rooted thinking of traditional Chinese medicine in workers' minds. In traditional Chinese medicine, the theory of yin and yang believes that the decline of the body's resilience leads to the imbalance of yin and yang and causes disease invasion [24]. Several studies on African residents have shown that people's wrong perception of malaria leads them to have inappropriate health behaviours, resulting in a higher malaria infection rate [25–27]. Therefore, it is imperative to increase the publicity of malaria from different channels, innovate publicity methods, and improve malaria awareness among Chinese workers in sub-Saharan Africa to reduce their malaria infection rate.

The study also discovered that many Chinese workers going to sub-Saharan Africa are still confused about malaria, including why some workers have never been infected with malaria, why one is still infected with malaria after protection, and what kind of people mosquitoes like to bite. This finding is also consistent with what Morgan and Figueroa-Munoz found in their study. It reported that there had been considerable debate in most groups about the observation that some people get malaria and others in a similar situation do not. Although there was no consensus, various explanations have been proposed, including blood type's influence and sickle cell disease [18]. Some other researchers have also answered some questions, such as the research by Zhao Jiawei and Del Marmol on the reasons why some people are more attractive to mosquitoes than others [28]. Some other confusion around mosquitoes still needs further studies. These confusions may reduce workers' self-efficacy in malaria prevention and control, and self-efficacy will be directly related to the malaria infection rate [29].

In addition, the study also found that Chinese construction workers interpreted malaria as a special meaning, like a "symbol of honor" and "predestined arrangement." Affected by the corporate culture, some workers, especially managers, think that people who have not been infected with malaria do not count as having been to Africa and even regard malaria as a sign of honor and hard work. Many workers believe that malaria infection in Africa is unavoidable, which is consistent with the research conducted by Newell et al*.* on residents in Peru [30]. These ideas will affect workers' attitudes towards malaria prevention and control behaviours and change their risk of infection. In future health education, the health sector needs to readjust the teaching focus and correct these deep-rooted views in workers' minds.

## Conclusion

China-Africa cooperation is deepening, and economies, trade, and people-to-people exchanges are growing closer. At the same time, malaria has become a severe health issue for Chinese workers in sub-Saharan Africa, who are at increased threat of infection and complications owing to an absence of immunity and exposure to high-transmission environments. One of the biggest challenges facing this category in fighting malaria is their lack of knowledge and misinterpretation, which can impact their intervention requirement, treatment adherence, and health services. These factors influence their preventive and therapeutic behaviors and health outcomes. Therefore, it is crucial to understand the perception and interpretation of malaria by Chinese workers in sub-Saharan Africa, as this may influence future health management strategies. This study adopts qualitative research methods to examine the understanding of malaria among Chinese construction workers in sub-Saharan Africa, which provides a source for future health management. Given the findings of this research, here are some possible recommendations. First, there is a requirement for workplace training programmes to provide accurate knowledge about the disease. These programmes could include information about malaria transmission, its symptoms, and the significance of prevention measures. Second, workplace health communication strategies must be evolved to counter rumours and offer accurate information. Third, additional research is essential to understand why some Chinese workers believe contracting malaria is a rite of passage for a stay in Africa. Understanding this perception could support developing approaches to change this belief. Finally, more transparent communication about the efficacy and shortcomings of various workplace prevention measures is needed to sidestep confusion about malaria infections.

## Data Availability

The datasets used and analyzed during the current study are available from the corresponding author upon reasonable request.

## Appendix

The question guide and probing questions:

1. What do you think about malaria?
  - Can you tell us more about why you feel like that?
  - Have your feelings changed over time?
2. How did your knowledge of malaria come about?
  - Can you recall any specific events or experiences that made you aware of malaria?
  - How does this awareness affect your daily life?
3. Do you think malaria can be prevented? How do you prevent malaria?
  - Can you share more details about ways to prevent malaria?
  - How effective do you think these methods are?
4. What was your experience during and after malaria infection?
  - Can you describe how you felt when you first realized you had malaria?
  - How does malaria affect your daily activities or rest?
5. What do you think about the dangers of malaria?
  - Can you explain why you think malaria is a danger?
  - Has this perception influenced your actions or decisions in any way?
6. What do you think causes malaria infection?
  - Can you elaborate on the factors that you believe contribute to the spread of malaria?
  - How do these factors affect your approach to malaria prevention?
