# Correction: The perception and interpretation of malaria among Chinese construction workers in sub-Saharan Africa: a qualitative study

**DOI:** 10.1186/s12936-023-04770-5

**Published:** 2023-11-03

**Authors:** Li Zou, Haohao Ma, Mohammad Shahir Sharifi, Wenyu Deng, Xiaoyu Kan, Junfei Luo, Yin Bai, Yunling Ouyang, Wenjuan Zhou

**Affiliations:** 1 School of Humanties, Central South University, Changsha, Hunan Province China; 2 School of Media and Communication, Shanghai Jiao Tong University, Shanghai, China; 3 Insurance Professional College, Changsha, Hunan Province China

**Correction: Malaria Journal (2023) 22:305** 10.1186/s12936-023-04739-4

Following publication of the original article [1], it came to the authors' attention that the affiliations information of the third author, Mohammad Shahir Sharifi, was incorrect: the author had been assigned affiliations 1 and 2 while they should just be affiliated with '2’. The published article has since been updated to correct this and the corrected affiliations information can be seen in this erratum. The authors thank you for reading and apologize for any inconvenience caused.
